# An Animal Model Using Metallic Ions to Produce Autoimmune Nephritis

**DOI:** 10.1155/2015/269610

**Published:** 2015-05-10

**Authors:** Roxana Ramírez-Sandoval, Nayeli Luévano-Rodríguez, Mayra Rodríguez-Rodríguez, María Elena Pérez-Pérez, Sergio Saldívar-Elias, Reinaldo Gurrola-Carlos, Esperanza Avalos-Díaz, Juan José Bollain-y-Goytia, Rafael Herrera-Esparza

**Affiliations:** ^1^Laboratorios de Inmunología y Biología Molecular, UA Ciencias Biológicas, Universidad Autónoma de Zacatecas, 98040 Zacatecas, ZAC, Mexico; ^2^Departamento de Patología, Universidad Autónoma de Durango, Campus Zacatecas, 98160 Zacatecas, ZAC, Mexico

## Abstract

Autoimmune nephritis triggered by metallic ions was assessed in a Long-Evans rat model. The parameters evaluated included antinuclear autoantibody production, kidney damage mediated by immune complexes detected by immunofluorescence, and renal function tested by retention of nitrogen waste products and proteinuria. To accomplish our goal, the animals were treated with the following ionic metals: HgCl_2_, CuSO_4_, AgNO_3_, and Pb(NO_3_)_2_. A group without ionic metals was used as the control. The results of the present investigation demonstrated that metallic ions triggered antinuclear antibody production in 60% of animals, some of them with anti-DNA specificity. Furthermore, all animals treated with heavy metals developed toxic glomerulonephritis with immune complex deposition along the mesangium and membranes. These phenomena were accompanied by proteinuria and increased concentrations of urea. Based on these results, we conclude that metallic ions may induce experimental autoimmune nephritis.

## 1. Introduction

Autoimmunity induced by metallic ions was previously described in experimental animals and humans suffering from chronic intoxication by heavy metals. However, the pathogenic mechanisms are only partially understood. One possibility is that interactions between metallic ions and normal proteins may trigger a self-immune-response characterized by autoantibody production and cell hypersensitivity. A second possibility is that stereochemical alteration of normal proteins results in the exposition of cryptic self-epitopes or neoepitope formation capable of activating autoreactive clones. A third possibility is related to the increased production of apoptotic remains, resulting in externalization of intracellular antigens onto apoptotic cell membranes. Regardless of how autoantigens are produced, they are processed and presented via MHC molecules to the TCR, resulting in polyclonal activation and autoimmunity [1].

Different examples of autoimmune responses triggered by ionic metals have been reported. For instance, cadmium ions induce autoantibodies to laminin, chromium and platinum trigger antinuclear antibodies (ANA), copper induces antibodies against red blood cells, lithium is associated with ANA and antithyroid antibodies, gold stimulates the production of ANA with Ro specificity and antiplatelet autoantibodies, and finally silver salts induce antifibrillarin antibodies [2]. As a consequence, it is widely accepted that metallic ions are potential inducers of autoimmune responses.

The kidneys are sensitive organs involved in autoimmune diseases, especially lupus erythematosus. Thus, this interesting topic deserves our attention. Previous works demonstrated that exposure to mercury was associated with autoimmune nephritis accompanied by autoantibody production and increased levels of proinflammatory cytokines such as TNF*α*, IL-1, and IFN*γ*. Similarly, animals that suffer contamination with mercury ions or their derivatives display membranous nephropathy [3–6].

In the present study, we induced autoimmune nephritis in inbred Long-Evans rats by intoxication with heavy metals; this model was chosen because other researchers previously reported nephrotoxicity in rats including Brown Norway (BN) and Sprague-Dawley; also lower doses of mercuric chloride administrated to outbreed Wistar rats resulted in renal toxicity [7]. We used inbred Long-Evans strains to maintain homozygosis; therefore, we observed in this particular strain high sensitivity to heavy metals injuries; therefore, this animal model was used to induce autoimmunity and we assessed antinuclear antibodies, kidney damage, and abnormalities in glomerular function. Main results of present work indicate that metallic ions can trigger glomerulonephritis associated with autoantibodies.

## 2. Material and Methods

### 2.1. Animals

Male Long-Evans rats that were 8 weeks of age with an average weight between 198 and 258 g were used for the present experiments. The animals were maintained under standard conditions at the animal facility unit of our research institute with free access to food and water. The animals were exposed to metallic ions by a weekly injection. Serum and urine samples were collected simultaneously with the weekly injections, and tissue biopsies were obtained at the end of the intoxication trial. The kidneys were collected immediately after sacrifice. Experiments were conducted according to the guidelines for ethical conduct in the care and use of animals developed by the American Psychological Association (APA) (http://www.apa.org/science/anguide.html).

### 2.2. Experimental Groups

Animals were grouped (*n* = 5) according to the treatments with metallic ions as follows: Group (I), the control group treated with 100 *μ*L of sterile 0.9% NaCl; Group (II) treated with HgCl_2_ (1.5 mg/100 g of weight); Group (III) treated with CuSO_4_ (3 mg/100 g of weight); Group (IV) treated with AgNO_3_ (3 mg/100 g of weight); and Group (V) treated with Pb(NO_3_)_2_ (6 mg/100 g of weight). The metallic ions were dissolved in distilled water and administered subcutaneously weekly over a period of 8 weeks. Urine and serum samples were obtained to determinate proteinuria and urea concentrations by conventional methods. At the end of the trial the animals were anesthetized with ether and sacrificed, followed by kidney excision. The organs were rinsed with PBS. One tissue fragment was included in Tissue-tek, frozen at −20°C, and then used for direct immunofluorescence. The other kidney fragment was fixed in 10% formalin and processed for H&E staining and immunohistochemistry.

### 2.3. Antinuclear Antibodies

Commercial slides containing HEp-2 cells (Immuno Concepts NA, Ltd, Sacramento, CA) were incubated for 30 minutes with serum diluted from 1 : 80 to 1 : 1280, followed by three times of PBS washing and a 30-minute incubation with FITC-labeled goat anti-rat polyvalent gamma globulin (IgG, IgA, and IgM; SAB3700662 Sigma). After another round of washing, the slides were mounted in glycerol-PBS and evaluated with fluorescence microscopy. And anti-DNA antibodies by immunofluorescence using* Crithidia luciliae* (Immuno Concepts NA, Ltd, Sacramento, CA). The fine specificity of sera samples was tested by ELISA against the following recombinant proteins: Ro60, La, and Sm/RNP (Euroimmun AG, Lübeck, Germany, and Orgentec Diagnostics GmbH, Mainz, Germany). Goat anti-rat polyvalent HRP-labeled anti-IgG and -IgM antibodies were used as the secondary antibodies (Sigma, SAB3700666-2MG).

### 2.4. Direct Immunofluorescence

A 4 *μ*m slice of renal tissue was obtained using cryosectioning. The tissues were rinsed in 0.15 M PBS, and any possible immune depositions were detected after incubation with FITC-labeled goat anti-rat polyvalent gamma globulin (IgG, IgA, and IgM; SAB3700662 Sigma). After 30-minute incubation, the slides were rinsed, mounted in glycerol-PBS, and evaluated using fluorescent microscopy.

### 2.5. Immunohistochemistry

To investigate intracellular autoantigen exposition in the kidneys, the tissues were tagged with monoclonal antibodies against the follow ribonucleoproteins: Sm (Pierce MA5-13449), RNP (Pierce MA1-34962), Ro60 (Santa Cruz Biotechnology, sc-100844), and La (Mybiosource MBS533624) as follows. Slides containing 4 *μ*m sections of renal tissue were dewaxed, permeabilized, and washed three times with PBS. Endogenous peroxidase was blocked with 3% horse serum inactivated at 56°C. After washing, the tissues were incubated for 1 hour with the monoclonal antibody diluted in 10% fetal calf serum/PBS according to the manufacturer's recommendations. The slides were washed with PBS and then incubated for 1 hour with HRP-labeled rabbit anti-mouse IgG. After washing, the color reaction was induced by 3,3′-diaminobenzidine-0.06% H_2_O_2_ (Sigma, Catalog number A9044), and finally the reaction was stopped with 2 N sulfuric acid. Assays were performed in duplicate and evaluated by two observers using a light microscope. The intensity of the color reaction obtained from kidneys by immunohistochemistry displays an intensity signal which was expressed in pixels and was analyzed with the software Image-Pro Plus Version 7.0. (Media Cybernetics, USA).

### 2.6. Double Fluorescence Labeling Assays

To assess the presence of intracellular antigens on apoptotic glomerular membranes, we colocalized the possible target proteins using a double fluorescence assay. Briefly, the autoantigens were tagged in red via 120-minute incubation with monoclonal anti-Sm and anti-RNP, anti-Ro60, or anti-La antibody. Next, the samples were incubated for 120 minutes with goat anti-mouse Texas red-labeled antibody (IgG-TR) (Catalog number sc-2781. Santa Cruz Biotechnology, Inc. Santa Cruz, CA). Apoptotic membranes were stained in green with FITC Annexin V (BD Pharmigen) (this assay is based on the property of cells to lose membrane asymmetry in the early phases of apoptosis because annexin V is a calcium-dependent phospholipid binding protein that binds with high affinity to phosphatidylserine). The tissues were rinsed with 1x binding buffer and then incubated for 15 minutes with 10 *μ*g of FITC-labeled recombinant annexin, followed by PBS washing. Finally, the slides were counterstained with DAPI, mounted, and evaluated under a fluorescence microscope using the appropriate filters for the dyes.

### 2.7. Podocyte Involvement

To investigate whether podocytes were affected by metallic ions, the tissues were blocked with 3% fetal bovine serum in PBS for 30 minutes and incubated for one hour with monoclonal anti-WT1 antibody (Catalog number sc-7385 Santa Cruz, Biotechnology Inc., Santa Cruz, CA) 1 : 100 in 10% FBS-PBS. After washing with PBS, the presence of the bound antibody was identified with goat anti-mouse IgG-TR. Additionally, following the washes some slides were counterstained with 4′,6-diamidino-2-phenylindole (DAPI). Finally, the slides were mounted and examined using fluorescent microscopy [8].

### 2.8. Statistical Analyses

The data were processed using nonparametric statistics (ANOVA and *t*-test) and chi-square test using Graph Pad Prism version 6.0 Software. A *P* value <0.05 was considered statistically significant.

## 3. Results

### 3.1. Kidney Pathology

The group of control animals did not develop any renal histology abnormalities. In contrast, the kidneys of animals treated with metallic ions developed tubular degeneration, glomerular swelling, and cell hypertrophy at the end of the trial. Furthermore, the urinary space was enhanced as a consequence of the glomerular atrophy, and many samples exhibited acute tubular edema and glomerular changes characterized by glomerular inflammation in varying degrees (Table 1).

**Figure 1:**
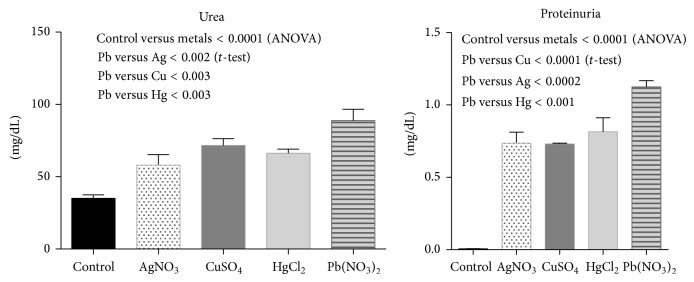
Urea and proteinuria determined at the beginning of the trial (control) and at the end of the intoxication trial. The graphs show the mean and standard deviation (SD).

The control group maintained normal renal function without proteinuria. The animals injected with different metallic ions developed renal dysfunction manifested by nitrogen waste product retention in all animals and in 50% of the animals display proteinuria at the second week but at the end of the trial all animals display proteinuria (Figure 1).

### 3.2. Autoantibody Production

**Figure 2:**
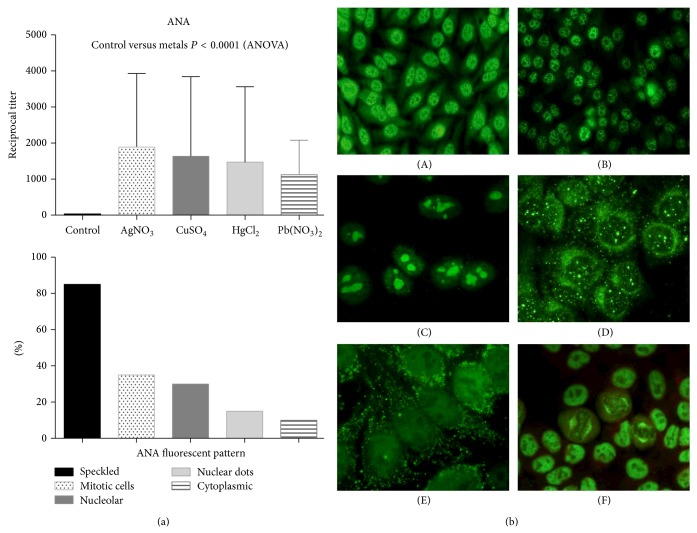
Indirect immunofluorescence assay graphs. (a) Antinuclear antibody (ANA) reciprocal titers using HEp-2 cells. The graphs show the mean and standard deviation (SD) and frequency of fluorescent patterns. And below is a graph showing the frequency of fluorescent patterns. On (b) different representative examples of ANA patterns: (A) nuclear speckled and cytoplasmic, (B) nuclear speckled, (C) nucleolar, (D) nuclear dots, (E) cytoplasmic, and (F) mitotic cells with staining of the nuclear mitotic apparatus.

**Figure 3:**
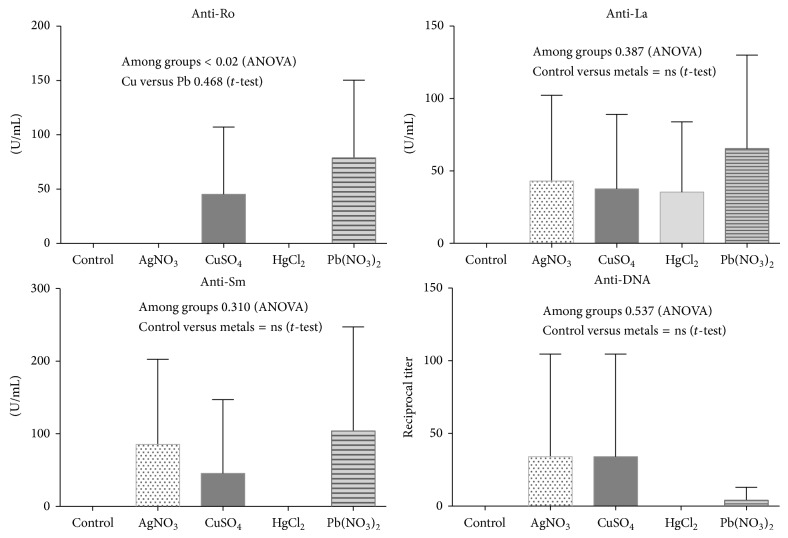
Antinuclear antibody specificity determined by ELISA (anti-Ro, anti-La, and anti-Sm) and the anti-DNA titers determined by indirect immunofluorescence with* Crithidia luciliae*. The graphs show the mean and SD.

Most of the animals displayed negative ANA titers at the beginning of the trial; however, all sera collected from animals treated with metallic ions tested positive for ANA determination four weeks after treatment, and the ANA titers increased at the end of the trial, and most of the ANA exhibited a cytoplasmic and speckled pattern. An interesting finding was that rats treated with mercury developed anti-nucleoli antibodies (Figure 2). Anti-La antibodies were ubiquitously present in all groups treated with different metallic ions, while anti-DNA and anti-Sm antibodies were ubiquitously present except in animals treated with HgCl_2_ (Figure 3).

### 3.3. Immune Complexes at the Glomerular Level

**Figure 4:**
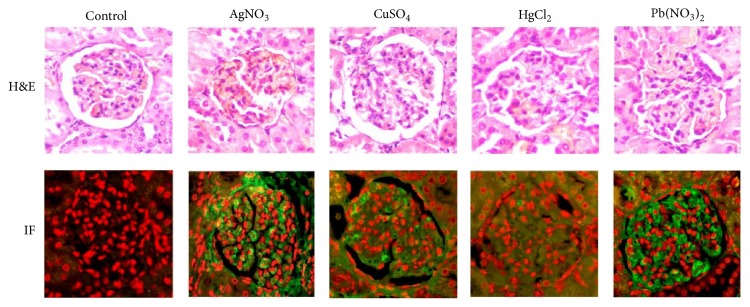
Renal histopathology. The top panel shows the control at the beginning of the trial and histology four weeks after each treatment by hematoxylin and eosin (H&E) staining. The bottom panel displays the glomerular direct immunofluorescence (IF) in the basal condition (control) and at the end of the trial. The immunofluorescence shows deposition of IgG in the glomerulus. Nuclei were counterstained using propidium iodide.

A total of 60% of treated animals showed localization of immune complexes (IC) in their kidneys. These complexes were composed of IgG in complex with antigens from mesangium and/or membranes. This finding was common in different groups of animals treated with different metals; however, the IC trapping in the glomeruli of animals treated with AgNO_3_ and Pb(NO_3_)_2_ was remarkable. In spite of the fact that immune complex deposition was demonstrated at glomerular level only in the 60% of animals, the renal damage was evident by histopathology in all animals treated with metallic ions; furthermore, all intoxicated animals exhibited increased levels of urea and proteinuria and positive antinuclear antibodies or immune complex immune deposition at tubuli regardless of the metallic ion used in the trial (Figure 4).

### 3.4. Renal Autoantigen

**Figure 5:**
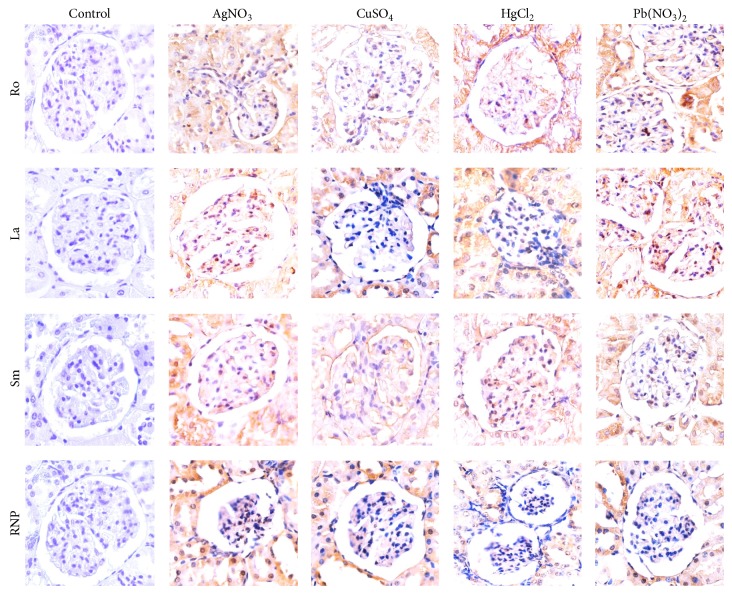
Immunohistochemistry of kidneys demonstrating the overexpression of ribonucleoproteins induced by heavy metal salts. Note that at the beginning of the trial (control), the expression is faint or absent, whereas at the end of the intoxication trial, ribonucleoprotein expression notably increases.

**Figure 6:**
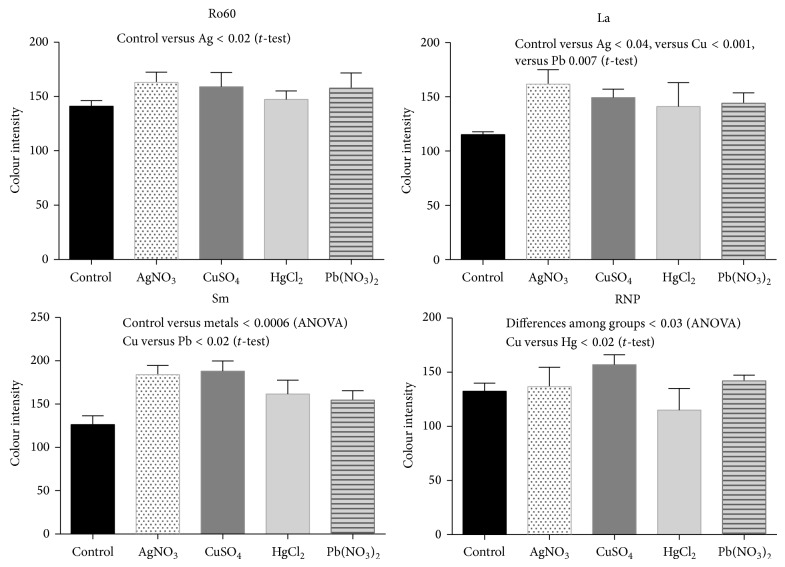
Ribonucleoprotein expression by immunohistochemistry of kidneys. The graphs (mean and SD) express the intensity of the color reaction obtained demonstrating the overexpression of some ribonucleoproteins induced by heavy metal salts.

**Figure 7:**
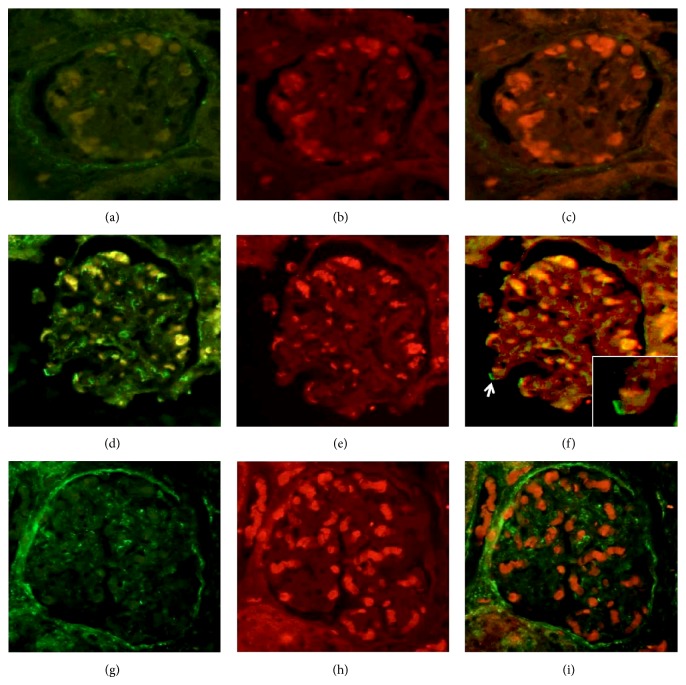
Double fluorescence assay showing apoptotic membranes of glomerular cells with annexin stained in green (a, d, and g) and intracellular autoantigens stained by Texas red (b, e, and h). The overlap merge is shown on the right (c, f, and i). In the upper panel (a, b, and c), a control without metallic ions is shown, characterized by the absence of annexin and the faint presence of intracellular Ro protein. The sample in the middle panel (d, e, and f) was collected from an animal treated with CuSO_4_ and shows in the overlap fine linear traces in green of apoptotic membranes of podocytes (arrow and the square magnification) close to the translocated Ro autoantigens. The inferior panel corresponds to an experimental animal treated with AgNO_3_. Interestingly, annexin staining is broadly distributed along the membranes of the Bowman capsule and mesangial area coincident with the Sm ribonucleoprotein.

**Figure 8:**
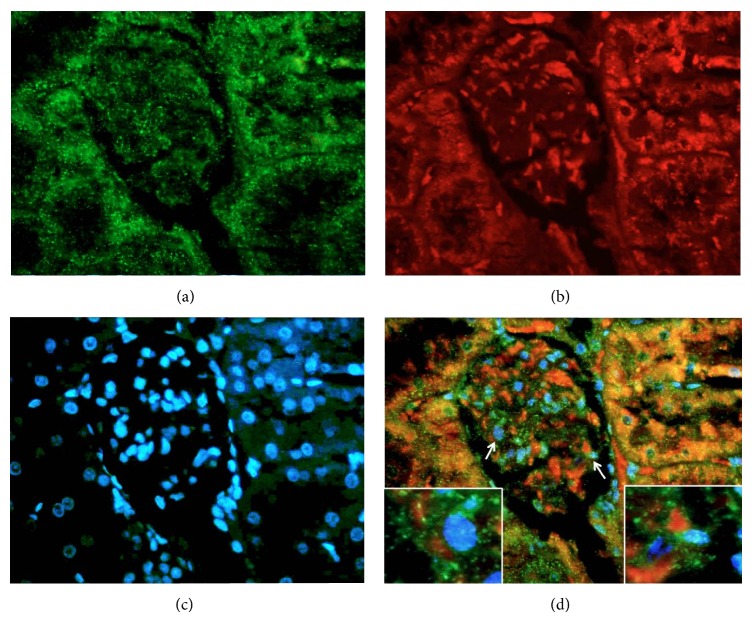
Double fluorescence assay in an animal treated with CuSO_4_ showing (a) a glomerulus stained in green by FITC-annexin localized to the apoptotic membranes, (b) La ribonucleoprotein stained by Texas red, (c) glomerulus nuclei stained in blue by DAPI, (d) merging image which shows colocalization of La/annexin; the arrows are representative examples of fields analyzed for colocalization of annexin (green) and La ribonucleoprotein (red) onto apoptotic membranes. The squares correspond to magnification areas pointed by arrows.

**Figure 9:**
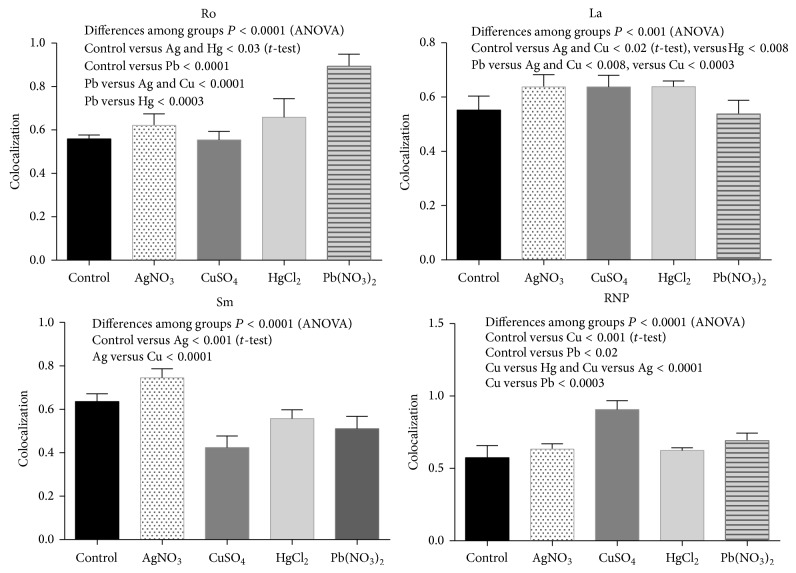
Colocalization analysis graphs (mean and SD) of ribonucleoproteins and apoptotic membranes (annexin) induced by heavy metal salts.

**Figure 10:**
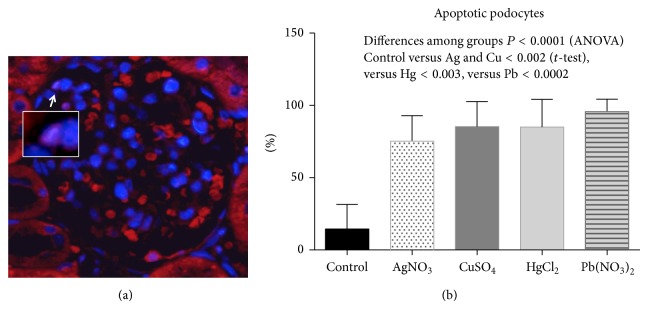
(a) Glomerulus showing podocytes tagged by WT1 in pink (arrow): the square corresponds to a magnified podocyte, nuclei stained in blue by DAPI. On (b) a graph showing the percent of apoptotic podocytes by metallic ions.

Two assays were performed to assess the expression and externalization effect induced by metals on the exposure of Sm, RNP, Ro, and La autoantigens on apoptotic membranes of glomerular and tubular cells: immunohistochemistry and double fluorescence. (a) All ribonucleoproteins were strongly expressed following intoxication with all tested metallic ions (Figures 5 and 6), whereas the Ro antigen was detected in animals treated with AgNO_3_ and Pb(NO_3_)_2_ and the Sm ribonucleoprotein was overexpressed in animals treated with CuSO_4_ and AgNO_3_. (b) Interestingly, the Ro and La autoantigens were distributed on the apoptotic membranes of mesangial cells and podocytes (Figures 7 and 8). Autoantigens are normally expressed inside of tubular and glomerular cells rather than externalized onto apoptotic membranes, as was the case for the animals treated with heavy metals (Figure 9). This type of apoptotic process involves the podocytes, as shown in the podocyte marker WT1 (Figure 10).

## 4. Discussion

Exposure to metal ions is the result of human activities such as mining, industrial processes, and other activities; this produce contamination of water sources, food, and consequently the inadvertent exposure or accidental contamination with heavy metals may affect humans or animals which can develop autoimmunity. The experimental models of autoimmunity induced by metal ions are important because they allow us to explore the pathophysiological mechanisms that trigger autoimmunity, and using this approach makes it possible to clarify clinically manifestations observed in human beings who are accidentally or inadvertently exposed to these pollutants.

The present study was undertaken to address the possible role of metallic ions in autoimmunity. Autoimmunity was triggered using an experimental rat model based on treatment with different heavy metals. The main findings can be summarized as follows. (1) Metallic ions such as mercury, lead, silver, and copper trigger the production of antinuclear antibodies. (2) The animals develop kidney damage demonstrated by proteinuria and urea retention. (3) Morphology changes including glomerular swelling and tubular degeneration were induced by the metals. (4) Immune glomerular damage was mediated by immune complexes. Taken together, we can conclude that heavy metals are capable of triggering autoimmune nephritis.

Experimental nephritis induced by mercury chloride [9], cadmium, and gold was previously reported [10–12]; additionally other reports suggest that lead might alter the clearance of immune complexes at the mesangial level [13]. In current investigation doses reported by other authors were used in the trial [14, 15], and our results agree with previous reports demonstrating that metallic ions may induce autoantibodies in serum and cause immune complex deposition along the mesangium and glomerular base membranes [16]. The original contribution of the present work demonstrates that the overexpression of intracellular ribonucleoproteins such as Ro, La, RNP, and Sm is caused by apoptosis; therefore, based on the results of our double fluorescence assays, we demonstrate that intracellular antigens are externalized onto the surface of apoptotic membranes and the externalization of autoantigen might contribute to disrupt the tolerance. Another original observation is that the toxic effect of ionic metals decreases the podocyte number and also modifies its diaphragm function resulting in proteinuria; this is a new finding that was not previously reported. Another important observation of this study indicates that homozygous animals are more susceptible to the nephrotoxic effects of metal ions; in this sense we previously found (unpublished observations) that heterozygous animals showed reduced susceptibility to renal injury by metal salts. The biggest limitation is the small number of animals included in each experimental group, which does not rule conceptual observation that metal salts induce apoptosis, helping to outsource autoantigens which triggers autoimmunity that favors the* in situ* immune complex formation which promotes renal failure and proteinuria.

In addition to the acute toxic effect of metallic ions on glomerular and tubular cells, it appears that subacute intoxication induces an autoimmune process in the kidneys. The mechanism responsible for this phenomenon that modifies the immune tolerance is still under investigation.

The effects of metallic ions on the immune response can be demarcated in at least two aspects. First, the metallic salts directly affect the conformation of proteins, resulting in autoantigen production; and, second, the adjuvant effect on the immune cells induces polyclonal cell activation. Both possibilities are mutual but not exclusive. A third factor involved in immunogenicity is the conformational changes that directly cause the metal ions on protein; so, it is important to understand that the metal binding sites on proteins are diverse and involve factors such as geometries, ligands, and metal preferences for certain protein domains. Nevertheless, metals commonly affect the highly hydrophobic centers [17]. Proteins with cysteine or histidine residues are modified by metal bonding and such interactions disrupt disulfide bonds or salt bridges. This type of disruption results in conformational changes in the secondary and tertiary structures, which can modify protein properties. Moreover, these stereochemical changes can expose cryptic antigens or induce the formation of neoantigens, resulting in better accommodation of “antigenic peptides” inside class II molecules of antigen presenting cells. These changes, together with the stimulation of autoimmune repertoires, render a breakdown of immune tolerance.

To predict the residues of autoantigens that are susceptible to metal binding, the Uniprot KB/Swiss-Prot data bank and the NCBI graphical sequence viewer were used to analyze metal-ion dependent adhesion sites (MIDAS) in the Ro60, La, Sm and RNP ribonucleoproteins. The Ro60 ribonucleoprotein (Uniprot KB/Swiss-Prot: P10155.2) displayed three binding sites at positions 378, and 380 (serine), and 445 (threonine), suggesting that there is a molecular basis to assume that Ro60 can be modified by metal bonding (Table 2). Thus, modification of its behavior as an autoantigen is theoretically possible based on our findings of the presence of anti-Ro antibodies in the serum and deposited along the glomeruli of experimental animals.

In contrast, the La, RNP, and Sm ribonucleoproteins did not display any MIDAS; however, this negative prediction did not rule out the possibility of stereochemical modification through interaction with –SH and carboxylate groups (which result in protein denaturation) as another potential source of antigenic peptides.

Metallic ions directly affect the immune system by working like an adjuvant. It is well known that the administration of subtoxic doses of mercury and other metals induces polyclonal activation of lymphocytes, yielding highly specific autoantibodies [3, 18].

In present work the cellular pathway involved in autoimmunity elicited by mercury salts encompasses endosomal TLR signaling; in turn, this signaling stimulates transcription factor NF-kB and promotes the transcription of the pro-IL-1B and IL-6 genes. Then, pro-IL-1B is activated by caspase 1 at the inflammasome, and both cytokines promote autoimmunity [19–21]. Additionally, the lymphocyte activation gene-3 (LAG-3) is an important regulator of autoimmunity in response to mercury [22].

## 5. Conclusions

In conclusion, this study focused on the analysis of environmentally induced autoimmunity using an animal model. Based on the present findings, we can suggest that metallic ions induce autoreactive clones that trigger autoantibody production against intracellular ribonucleoproteins. These ribonucleoproteins are then exposed on apoptotic membranes, enabling the formation of immune complexes along the glomeruli. Based on this experimental approach, we conclude that metallic ions induce autoimmune nephritis.

## Figures and Tables

**Table 1:** Major histological findings in animals intoxicated with metal ions.

Control	Glomeruli conserved, tubules, interstice, and blood vessels normal

AgNO_3_	Glomeruli with mesangial moderate hypercellularity, interstitial lymphocyte infiltration, congested blood vessels, edema of convoluted tubules, and the presence of proteinaceous material

CuSO_4_	Glomerular size increased as Bowman space decreased; podocytes with enlarged volume, thickened membranes, and discrete lymphocytic infiltrates, tubular edema with the luminal presence of proteinaceous material, and diffuse vascular congestion

HgCl_2_	Glomerulus and Bowman space enlarged, mesangial cell proliferation, podocytes with increased volume, increase in membrane thickness, interstitium with inflammatory infiltrate of neutrophils, and tubules with epithelial edema and presence of acidophilus material

Pb(NO_3_)_2_	Glomeruli with slight increase in size, increased Bowman space, increased membrane thickness, discrete inflammatory infiltrate, and tubular edema

**Table 2:** Ro60 ribonucleoprotein analyzed using the NCBI graphical sequence viewer displaying the three metal-ion dependent adhesion sites (MIDAS) at positions 378, and 380 (serine), and 445 (threonine) (UniProtKB/Swiss-Prot: P10155.2).

P10155[455], 60 kDa SS-A/Ro ribonucleoprotein, Homo sapiens					
10 MEESVNQMQP	20 LNEKQIANSQ	30 DGYVWQVTDM	40 NRLHRFLCFG	50 SEGGTYYIKE	60 QKLGLENAEA
70 LIRLIEDGRG	80 CEVIQEIKSF	90 SQEGRTTKQE	100 PMLFALAICS	110 QCSDISTKQA	120 AFKAVSEVCR
130 IPTHLFTFIQ	140 FKKDLKESMK	150 CGMWGRALRK	160 AIADWYNEKG	170 GMALALAVTK	180 YKQRNGWSHK
190 DLLRLSHLKP	200 SSEGLAIVTK	210 YITKGWKEVH	220 ELYKEKALSV	230 ETEKLLKYLE	240 AVEKVKRTRD
250 ELEVIHLIEE	260 HRLVREHLLT	270 NHLKSKEVWK	280 ALLQEMPLTA	290 LLRNLGKMTA	300 NSVLEPGNSE
310 VSLVCEKLCN	320 EKLLKKARIH	330 PFHILIALET	340 YKTGHGLRGK	350 LKWRPDEEIL	360 KALDAAFYKT
370 FKTVEPTGKR	380 FLLAVDV**S**A**S**	390 MNQRVLGSIL	400 NASTVAAAMC	410 MVVTRTEKDS	420 YVVAFSDEMV
430 PCPVTTDMTL	440 QQVLMAMSQI	450 PAGG**T**DCSLP	460 MIWAQKTNTP	470 ADVFIVFTDN	480 ETFAGGVHPA
490 IALREYRKKM	500 DIPAKLIVCG	510 MTSNGFTIAD	520 PDDRGMLDMC	530 GFDTGALDVI	RNFTLDMI
	**Divalent metal cation**				
